# Grand challenge in microbiome data science: recovering the microbiome as a system

**DOI:** 10.3389/fmicb.2026.1970739

**Published:** 2026-09-09

**Authors:** Peter Belenky, Folker Meyer

**Affiliations:** 1 Department of Molecular Microbiology and Immunology, Brown University, Providence, RI, United States; 2 Department of Medicine and Department of Computer Science, University of Duisburg-Essen, Essen, Germany

**Keywords:** artificial intelligence, causal inference, FAIR data, metagenomics, microbiome data science, multi-omics, reproducibility, systems biology

## Introduction

The microbiome has never been only a census of organisms. In its original ecological formulation, the term described a microbial community embedded in a defined habitat, with its own physicochemical conditions and “theater of activity” (Berg et al., 2020; Whipps et al., 1988). That original framing is useful now because it reminds us that microbes do not act as isolated names on a taxonomic list. Rather, they act in place, in communities, through metabolism, interaction, adaptation, and response to their host or environment.

Sequencing transformed the field by making microbial communities measurable at scale. For practical reasons, however, much of early microbiome analysis reduced this complexity to taxonomic profiles and relative abundance tables. That reduction gave the field its first maps but also created a gap between what microbiomes are and what our standard data structures can easily represent. The grand challenge for microbiome data science is to close that gap: to turn increasingly diverse measurements into knowledge that is reproducible, interpretable, mechanistic, and actionable. In this Grand Challenge article, we argue that microbiome data science must become the framework that allows microbiomes to be studied as biological systems.

## Defining the scope of microbiome data science

To define this space, microbiome data science must be understood not as a single method or data type, but as the framework that connects measurement, computation, and interpretation across microbial systems. Its scope extends beyond individual samples. It includes comparative studies of microbiomes across individuals, habitats, time, locations, interventions, and disease states, along with the residual fingerprints that microbiomes leave in shared environments, such as rivers, oceans, drinking water systems, built environments, and wastewater. The field is therefore not drawing a single map but rather the outlines of many microbial continents whose boundaries shift with host state, ecology, technology, and scale.

This scope is expanding because measurement is becoming cheaper, faster, and less dependent on cultivation. Genomic and multi-omic methods now make it possible to chart multiple microbial habitats within one person, compare niches such as the gut, airway, skin, or wound microbiome, and follow community change under therapy, infection, nutrition, or environmental factors.

Microbiome data can describe ecosystem state and health status, but its larger promise is predictive and generative. Historically, many analyses were organized around predefined hypotheses and a limited set of features. As cohorts, longitudinal sampling, and environmental surveillance expand, data science can reveal previously unrecognized ecological states, transitions, and potential mechanisms that generate experimentally testable hypotheses. This changes the role of computation from confirming associations to determining what should be measured or perturbed next.

## From taxonomic description to functional and mechanistic inference

A central challenge for microbiome data science is to move from functional annotation to functional understanding. Marker-gene surveys and early metagenomic projects made microbial communities measurable; however, they also exposed a persistent problem: knowing which organisms are present does not tell us what the community is doing. The Human Microbiome Project showed this clearly, revealing that taxonomic variation among healthy individuals often exceeds variation in metabolic pathway carriage (Huttenhower et al., 2012). Organism-resolved profilers, such as HUMAnN2 (Franzosa et al., 2018) and the bioBakery3 tools (Beghini et al., 2021), connected reads to gene families, pathways, strains, and contributing taxa, while co-abundance gene groups identified ecological and functional units that are not fully captured by reference genomes (Minot and Willis, 2019; Nielsen et al., 2014).

Yet these challenges remain unresolved at several levels. The majority of microbial genes are still poorly characterized. Pathway presence does not guarantee pathway activity. Community function often emerges from interactions among organisms rather than from any single genome. Metabolic reconstructions, such as AGORA2, showed how strain-resolved composition can be connected to predicted biochemical outputs, including drug biotransformation; however, this is only part of the path forward (Heinken et al., 2023). The next phase must integrate reference-based profiling, reference-light discovery, expression and metabolite data, causally informative experiments, and community-scale modeling (Zhang et al., 2025; Quinn-Bohmann et al., 2025). The goal is not simply to annotate more genes. It is to build models that explain how microbial communities generate biochemical activity in context.

## Study design, measurement, and trustworthy inference

Trustworthy microbiome data begin before sequencing. Sampling strategy, storage, extraction, library preparation, batch structure, sequencing depth, negative controls, mock communities, host metadata, and environmental covariates are all part of the experiment and not merely metadata attached afterward. Microbiome studies often seek effects that are small relative to the variation introduced by geography, diet, medication, sampling time, hospital practice, laboratory protocol, or contamination. Protocol-specific biases and reagent contamination can be systematic and reproducible, which makes them particularly dangerous because they can masquerade as biology (Costea et al., 2017; Salter et al., 2014; McLaren et al., 2019). With large datasets, statistical power does not rescue poor design; it can instead make artifacts highly significant.

The grand challenge is therefore to match design to inference. Cross-sectional studies can generate hypotheses, but state transitions, resilience, treatment response, and early warning require longitudinal sampling at a cadence compatible with microbial dynamics. Measurement models must account for compositionality, sparsity, absolute load, detection limits, and uncertainty in the mapping of reads to features (Gloor et al., 2017). Host and environmental variables can confound apparent disease associations and must be modeled explicitly rather than treated as an afterthought (Vujkovic-Cvijin et al., 2020). The decisive question is not whether an association is significant in a table, but whether it survives negative controls, protocol and batch variation, appropriate cohort separation, and independent validation, and whether it generates a laboratory-testable hypothesis that advances biological understanding.

## Multi-omics integration as the central bottleneck

A central challenge in microbiome data science is integrating the different layers through which microbial function becomes visible. No single measurement is sufficient. The metagenome defines the community's genetic capacity: the organisms, genes, and pathways that are present. Metatranscriptomic and metaproteomic data show which parts of that capacity are being expressed or executed. Metabolites provide the chemical readout of these activities, capturing what is produced, consumed, modified, or exchanged. These layers form a functional triangle of sorts. When they point in the same direction, they can strengthen inference. When they disagree, the disagreement itself can be informative, revealing information about regulation, inactive pathways, host modification, dietary inputs, or cross-feeding between organisms.

Longitudinal multi-omic studies have shown why this matters. Disease-associated microbiomes are not simply altered taxonomic states but rather dynamic molecular states involving microbial transcription, metabolites, immune features, diet, and time (Lloyd-Price et al., 2019). However, the field still lacks a common strategy for turning layered measurements into mechanisms rather than parallel lists of associated features. Multi-omic modules and metagenome-informed metaproteomics are important steps because they preserve relationships among species, pathways, metabolites, proteins, exposures, and phenotypes (Muller et al., 2024; Valdés-Mas et al., 2025). The grand challenge is to use these layers to triangulate function, deciding which measurements are needed for a given question, preserving temporal and spatial structure, and separating causal relationships from coincident correlations. The goal is not to build bigger matrices. It is to explain how microbiomes change state.

## Machine learning and AI for microbiome discovery: prediction is not enough

Machine learning is becoming central because microbiome data are high-dimensional, sparse, compositional, and heterogeneous. It can support diagnosis, risk stratification, source tracking, outbreak detection, phenotype prediction, feature discovery, and the integration of sequencing data with clinical or environmental metadata. However, prediction alone is insufficient. Cross-study analyses and microbiome classification benchmarks show how strongly apparent performance can depend on cohort structure, feature processing, and validation design (Pasolli et al., 2016; Topçuoglu et al., 2020). A model that accurately classifies disease status, hospital site, or wastewater origin may still have learned batch, diet, medication, sequencing center, or geography rather than transferable biology.

The grand challenge is to make AI robust, interpretable, and biologically constrained. Training and test data should be separated by cohort, time, site, or patient whenever those units could leak information; calibration and uncertainty should be reported; external validity should be tested; and explanatory features should be resolved at actionable levels, such as strains, pathways, mobile elements, metabolites, or ecological states. Foundation models for microbial genomes and sequences are now technically credible (Dalla-Torre et al., 2025), but they should be judged against transparent baselines and evaluated for transportability rather than novelty alone. The strongest model will be one that predicts across contexts and helps explain why the system changed.

AI should therefore function as a hypothesis engine rather than an oracle. To be useful for biological, clinical, or surveillance questions, a model should expose its dominant drivers, uncertainty, and failure domains, and ideally identify the next measurement or perturbation that would discriminate between competing explanations. In this sense, interpretability is experimental: a model becomes scientifically valuable when it produces a prediction or mechanism that can be falsified.

## Systems biology, causality, and perturbation-response models

Microbiomes are adaptive systems. They respond to antibiotics, diet, inflammation, infection, pH, oxygen, immune pressure, host tissue damage, phages, and nutrient flow. Their behavior is often nonlinear: small perturbations can be buffered, amplified, or redirected through cross-feeding, competition, horizontal gene transfer, or niche replacement. Similar taxonomic configurations can support different functions, while different communities may converge on similar biochemical outputs. Therefore, systems biology is needed because it treats the microbiome not as a static list of taxa, but as a network and state space of interacting organisms, genes, metabolites, and host or environmental constraints.

The next step is to connect observation with perturbation. Longitudinal data can demonstrate that a community changed before disease or environmental deterioration; however, temporal ordering alone does not establish the mechanism. Perturbation-response models are needed to determine whether the change was causal, compensatory, or correlated with another driver. Antibiotic exposure, diet, infection, transplantation, synthetic communities, bioreactors, organoids, animal models, and natural experiments can all constrain causal models. The aim should not be to assign a causal label through observational machine learning but rather to combine temporal structure, mechanistic priors, and perturbation evidence to learn state spaces, tipping points, recovery trajectories, and intervention targets.

## New measurement scales: spatial, single-cell, strain-resolved, virome, and mobile-element data

A central challenge in microbiome data science is that even well-integrated omics can miss biology if the sample has been averaged at the wrong scale. A stool sample, biopsy, or bulk metagenome collapses organisms across space, cells across physiological states, strains across genetic backgrounds, and viruses across transient host interactions. Spatial approaches make location a functional variable by mapping microbial biogeography together with host transcriptional state (Lötstedt et al., 2024; Ntekas et al., 2026). Single-cell approaches reveal minority states, stress responses, persisters, and metabolically specialized subpopulations that bulk profiles can dilute (Pountain and Yanai, 2025; Xu et al., 2023; Zheng et al., 2022). Virome, mobilome, and long-read methods add another layer by showing that phages, structural variation, recombination, methylation, and resistance-associated cargo are part of community function rather than background noise (Lai et al., 2024; Camarillo-Guerrero et al., 2021). The challenge is no longer just to measure more deeply. It is to build models that hold spatial organization, strain structure, cell state, viral ecology, and mobile genetic exchange together without flattening away the biology.

## Benchmarking, standards, reproducibility, and reanalysis-ready microbiome data

Microbiome data science cannot mature without stronger benchmarking and standards. Results still depend on database version, marker choice, assembly strategy, classifier, parameter setting, and preprocessing convention. Two pipelines may produce different taxonomic names, gene counts, pathway calls, or resistance profiles from the same reads, and both may look plausible unless tested against defined truth sets. Community-wide benchmarking efforts such as the Critical Assessment of Metagenome Interpretation (CAMI) have shown both the value of common datasets and the persistent trade-offs among methods (Sczyrba et al., 2017; Meyer et al., 2022). Benchmarking should cover the complete chain, from sampling and extraction to read processing, assembly, binning, annotation, normalization, statistical modeling, and interpretation.

Standards are not administrative overhead. They are what make microbiome findings comparable, reproducible, and reusable. MIxS-style contextual metadata (Yilmaz et al., 2011), persistent identifiers, containerized workflows, versioned reference databases, executable provenance, public benchmark datasets, and the FAIR principles (Wilkinson et al., 2016) are essential for cumulative science. For microbiome data, the FAIR principles should be complemented by a practical requirement: analyses should be reanalysis-ready. Raw measurements must remain linked to rich metadata, laboratory protocols, controls, software and database versions, parameters, intermediate feature definitions, and explicit uncertainty. Reproducibility should include future reproducibility: when a taxonomy, genome catalog, or annotation database changes, the analysis should be rerunnable, and the resulting differences should be explainable.

## Infrastructure, interoperability, privacy-preserving analysis, and human-in-the-loop tools

A central challenge for microbiome data science is that infrastructure determines what can be known. Microbiome results are shaped by sample context, reference databases, software versions, parameters, normalization choices, and intermediate feature construction; without this information, the same dataset may be impossible to reinterpret or combine. Shared analysis infrastructures, such as MG-RAST, have demonstrated early that public data become substantially more useful when deposition is coupled with standardized computation and comparative analysis (Meyer et al., 2008). Building on standards for metadata and provenance, the next infrastructure challenge is interoperability at scale: cross-cohort harmonization, privacy-aware or federated analysis, versioned resources, batch-aware modeling, and human scientific judgment. Infrastructure should be judged by whether it makes microbiome data easier to compare, reuse, refute, audit, integrate, and interpret, not by whether it adds another isolated database, workflow, or visualization layer. Otherwise, distributed models may learn the clinic, geography, or batch instead of biology.

Infrastructure also depends on resources that the field did not build itself: the underlying sequence and annotation repositories that all microbiome analyses draw on. These databases are not static; taxonomies are revised, genomes are added, and annotations drift or conflict across resources, often invisibly to those who rely on them downstream. The curators who maintain and reconcile these annotations are as much a part of microbiome infrastructure as any pipeline or workflow, and sustaining that curation work, not just building new microbiome-specific layers on top of it, should be treated as a core priority for the field.

## Discussion: recovering the microbiome as a system

This article should catalyze a shift in emphasis. Microbiome data science should not be judged only by larger cohorts, deeper sequencing, more features, or more powerful algorithms. It should be judged by its ability to help recover the microbiome as a system: spatially organized, temporally dynamic, functionally integrated, experimentally durable, and embedded in host and environmental context. This standard moves the field from cataloging components toward explaining state and state change.

Four priorities follow. First, experimental design, controls, and uncertainty must be treated as part of the computational model rather than as preprocessing details. Second, predictive models must be evaluated for transportability and causal utility rather than just discrimination within a cohort. Third, function should be inferred by triangulating metagenomic, expression, metabolite, spatial, single-cell, strain, viral, and perturbation information rather than from abundance alone. Fourth, every major claim should remain connected to reusable data, software, reference versions, and provenance so that it can be challenged and reinterpreted as knowledge changes.

Success would change the unit of microbiome data science from isolated features to biological state transitions and their mechanisms. In clinical settings, this could mean recognizing loss of resilience or treatment response before a static endpoint is reached; in environmental and public health surveillance, it could mean detecting coherent ecosystem change before conventional indicators become obvious. In both cases, action must remain proportional to the evidence and its uncertainty. The field will have matured when it reliably connects measurement to mechanism, prediction to validation, and data reuse to cumulative knowledge.
